# Process Monitoring Dataset from the Additive Manufacturing Metrology Testbed (AMMT): Overhang Part X4

**DOI:** 10.6028/jres.125.027

**Published:** 2020-09-03

**Authors:** Brandon Lane, Ho Yeung

**Affiliations:** 1 National Institute of Standards and Technology, Gaithersburg, MD 20899, USA

**Keywords:** additive manufacturing, laser powder bed fusion, selective laser melting

## 1 Summary

This document provides details on the files available in the dataset “Overhang Part X4” pertaining to a three-dimensional (3D) additive manufacturing (AM) build performed on the Additive Manufacturing Metrology Testbed (AMMT) by Ho Yeung and Brandon Lane on June 28, 2019. The files include the input command files, materials data, in-situ process monitoring data, and metadata. This data is one of a set of “AMMT Process Monitoring Datasets”, as part of the Metrology for Real-Time Monitoring of Additive Manufacturing project at the National Institute of Standards and Technology (NIST). Ex-situ part characterization data, including X-ray computed tomography (XCT) measurements, will be provided as it is made available. Readers should refer to the AMMT datasets web page for updates.

## 2 Data Specifications

**Table:** 

**NIST Operating Unit(s)**	Engineering Laboratory, Intelligent Systems Division
**Format**	There are several data formats in this dataset. Please refer to Sec. 4 for a detailed description of all included data files.
**Instruments**	The Additive Manufacturing Metrology Testbed (AMMT) was used to perform the laser scans. A Mikrotron EOSens 3CL^1^ camera was used for co-axial melt pool monitoring, and a Basler acA3800-10gm camera was used for layer-wise monitoring. Details are provided in Sec. 3.
**Spatial or Temporal Elements**	These measurements were performed on June 28, 2019
**Data Dictionary**	N/A
**Accessibility**	All datasets^2^ submitted to *Journal of Research of NIST* are publicly available.
**License**	https://www.nist.gov/director/licensing

1 Certain commercial equipment, instruments, or materials are identified in this paper in order to specify the experimental procedure adequately. Such identification is not intended to imply recommendation or endorsement by the National Institute of Standards and Technology, nor is it intended to imply that the materials or equipment identified are necessarily the best available for the purpose.

2 The National Institute of Standards and Technology (NIST) uses its best efforts to deliver a high-quality copy of the Database and to verify that the data contained therein have been selected on the basis of sound scientific judgment. However, NIST makes no warranties to that effect, and NIST shall not be liable for any damage that may result from errors or omissions in the Database.

## 3 Experiment Overview

This 3D build experiment was designed to create four nominally identical parts within the same build, where material, scan strategy, and laser processing parameters were replicated. The general intention is to determine if results of various analyses of process monitoring data conducted on one part can then be tested and compared against nominally identical, but different parts. Data include commanded scan strategy and laser parameters, co-axial melt pool monitoring (MPM) images, layer-wise imaging, and laser position and power feedback monitoring.

Various potential analyses are proposed:

1) General process monitoring analyses (e.g., signal or image processing, data visualization and registration, etc.);
2) Sensor to sensor combination or comparison (i.e., sensor fusion. Identify if features in one sensor relate to another);
3) Part to part comparison (e.g., identify if features or metrics extracted from sensors are the same or different between each part); and
4) In-situ process signature to ex-situ part quality comparison (e.g., defect identification).

Further ex-situ characterization of the parts, including XCT data and pore/defect characterization, will be provided in the future. Additionally, a second 3D build and corresponding process monitoring dataset will be provided in the future, which includes the same part geometries, materials, and scan strategies. Readers should refer to the AMMT datasets web page for updates. The experiment design is intended to provide training, validation, and testing data for development of data-driven predictive models.

**Fig. 1:**
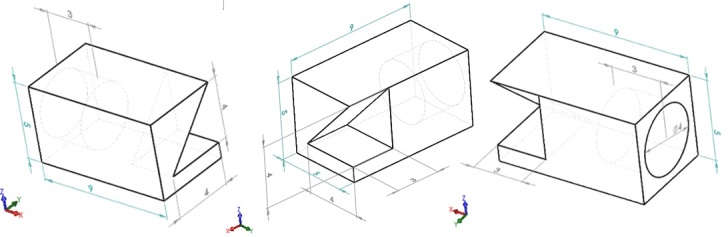
Three views of the external part geometry of the overhang part (units in [mm]).

The part geometry is shown in multiple views in Fig. 1. This geometry was designed as part of an earlier study conducted on a commercial laser powder bed fusion (LPBF) system with concurrent in-situ thermographic measurement of the melt pool [1]. Though similar in geometry, the scan strategy described in this paper is primarily composed of X and Y oriented scans (90° rotations) between each layer, whereas 67° rotations between each layer were used in [1].

**Fig. 2:**
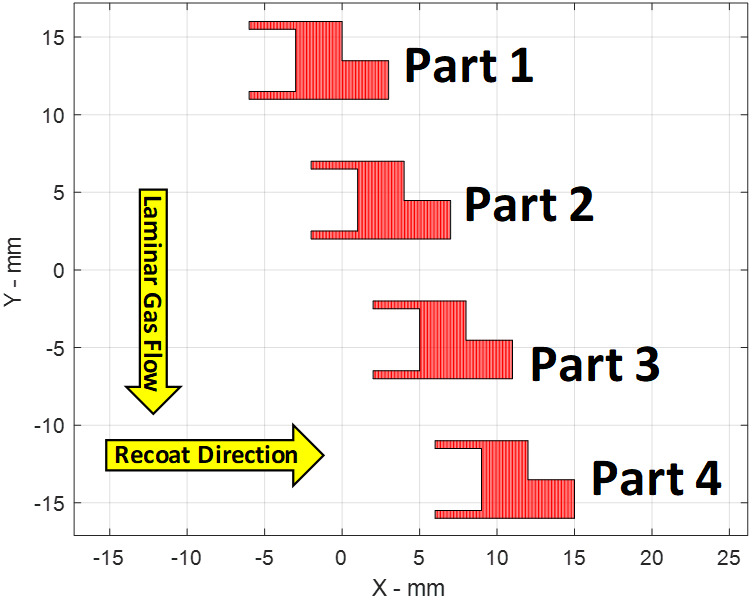
Part numbering and layout for Layer 125 of 250.

The placement and numbering of the four parts on the build plate are plotted in Fig. 2 at layer 125 of 250. This layer intersects the horizontal cylindrical cavity on the left (-X) side of each part, and the overhang feature on the right (+X) side of each part. The scan sequence proceeds from Part 1 to Part 4 in order, with each part being complete before moving to the next layer.

The build consists of 250 layers at 20 μm per layer. It was conducted in an inert argon gas environment (oxygen < 500 ppm), with laminar flow over the build plane. Gas flow velocity is unknown at the time of this publication, though gas flow rate was set to 300 L/min. The laminar flow exhaust nozzle has a 100 mm by 20 mm cross-section, which results in a calculated maximum 2.5 m/s flow velocity. The 3D build occurs within a smaller plexiglass build enclosure within the AMMT vacuum chamber that intercepts the laminar flow nozzle and exhaust, which is shown along-side other components in Fig. 11.

## 3.1 Process Monitoring Systems and Coordinate System Definitions

**Fig. 3:**
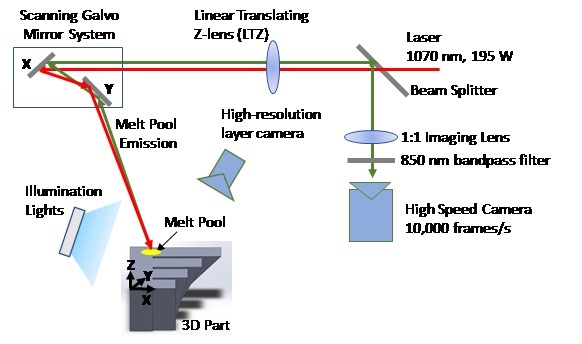
Schematic of the AMMT 3D build process monitoring systems, including the high-resolution layer camera (layer-wise imaging) and high speed MPM camera coaxially aligned with the laser. Figure reproduced from [2].

The following is reproduced from [2]:

“Data from two primary process monitoring instruments are acquired during the 3D build: the co-axial melt pool monitoring (MPM) camera, and the layer camera. Figure 3 shows a general layout of the laser, galvos, MPM camera, and layer camera. The co-axial MPM camera is optically-aligned with the laser axis, such that the melt pool appears stationary in the field of view regardless of galvo XY position. The layer camera is at a fixed position above the build plane.”

**Fig. 4:**
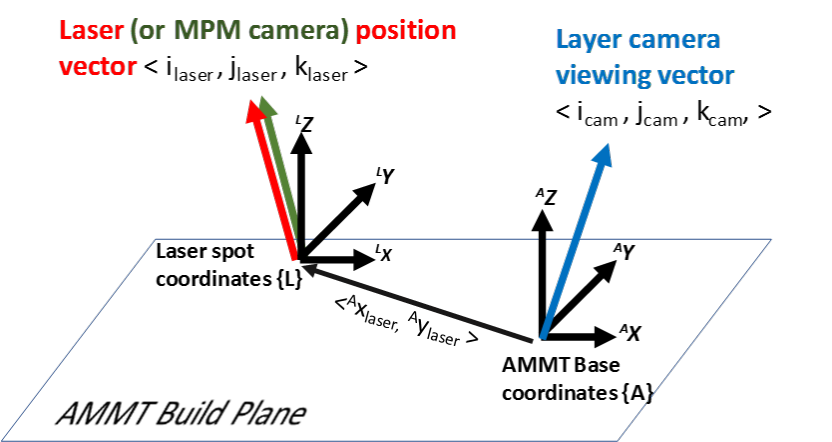
Identification of coordinate systems and descriptive vectors. Orientation of stationary instruments (such as the layer camera) can be defined as a unit vector in the AMMT base coordinate system {A}. The laser spot position in XY is defined by the vector <^A^x_laser,_^A^y_laser_ > in the AMMT base coordinate system {A}. Laser angle/orientation can be defined by a unit vector in the laser spot coordinate system {L}, which also defines the viewing angle of any co-axial instrument. Figure reproduced from [2].

“To describe the orientation and positions of the co-axial MPM camera, or stationary instruments such as the layer camera, the following coordinates are defined and shown in Fig. 4. The laser spot position is defined by the vector <^A^x_laser,_^A^y_laser_ > in the AMMT base coordinate system {A}. A translating coordinate system {L} is tied to the laser spot with x, y, and z axes aligned with those of {A} (pure translation, no rotation).”

## 4 Process Inputs: Materials, Geometry, and Build Command Files

### 4.1 Materials

Materials data is provided within the “BuildMetadata.zip” folder. The substrate material (on which the part is printed) is a wrought nickel alloy 625 (IN625) plate, hot rolled and annealed, and cut to 100 mm × 100 mm × 12.5 mm thick. The powder material was a recycled IN625, mixed from several lots of one-time-used and sieved powder. No additional powder information is given as part of this dataset, although composition of the virgin state of the IN625 powder can be found in [2]. The powder size distribution was measured using a dynamic imaging-based particle size analyzer for three samples taken from the powder bucket prior to the 3D build. Particle size distribution (PSD), calculated as the average of the three sample’s distributions based on Q_3_ volume metric, were D_10_ = 17 µm, D_50_ = 29 µm, and D_90_ = 46 µm.

Full particle size and morphology measurement results are provided in tab-delimited text files “PowderChar_X.txt”, where X is the sample number. An additional information sheet (MS Word document) defining the particle size metrics is also provided as “PowderCharacteristicsInfo.docx”. All powder data is provided in the Build Metadata>Powder subfolder.

### 4.2 Build Metadata

**Fig. 5:**
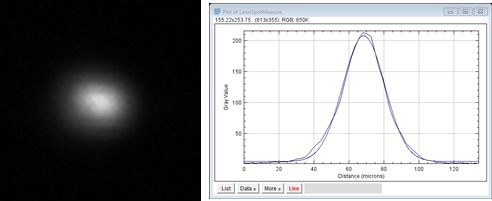
Left: Laser spot image. X and Y image axes are aligned with machine coordinate system {A}. Right: Example Gaussian curve fit to horizontal profile (σ = 14.92 μm, D4σ = 60 μm).

Multiple additional files pertaining to the system setup and materials are provided in the “BuildMetadata.zip” folder. “LaserSpotMeasure70x70.tif” provides a tagged-image file (TIF or TIFF) representing a measure of the laser beam spot profile used during the 3D builds, and is shown in Fig. 5. This was measured at the nominal (0,0) commanded galvo position. Pixels in this image are 2.2 μm square, and this scaling is also embedded in the TIF file. Orientation of the laser spot image is aligned with the base machine coordinate system{A}, and the laser coordinate system{L} center (refer to Fig. 4) can be assumed to be at the maximum grayscale value in the laser spot image. D4σ laser spot diameter, which is estimated as 4σ of a Gaussian curve fit to the measured laser spot intensity profile, was 60 μm in X and 48 μm in Y.

Laser angle as a function of X and Y position is provided in “Build Metadata.zip”. The following excerpt is reproduced from [2], which provided the same associated laser angle data file, with figure numbers changed for this document.

**Fig. 6:**
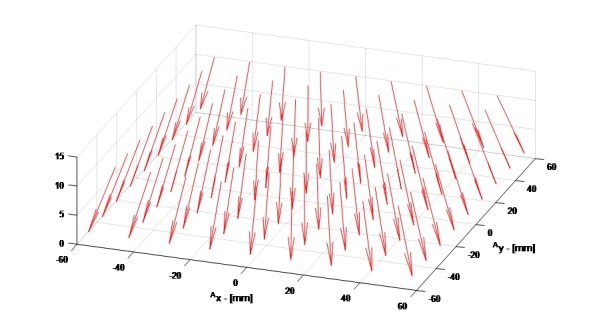
Laser orientation < i_laser_, j_laser_, k_laser_ >, mapped to the corresponding ^A^$\vec{r}$_laser_ position in the AMMT base coordinates. The i_laser_ and j_laser_ magnitudes are amplified 5x in this plot to highlight the laser angle (from [2]).

“2018_AMMTLaserScanAngles.txt” is a tab-delimited text file that provides the laser incident angle unit vectors with respect to the build plane as shown in Fig. 4. Three tables, representing i_laser_, j_laser_, and k_laser_ vector components, are provided vs. laser positions in the AMMT base coordinate system, where the top row and left column are the positions of ^A^$\vec{r}$_laser_ in [mm]. Figure 6 gives an example quiver plot showing the results, where i_laser_ and j_laser_ magnitudes are amplified 5x to demonstrate how the laser angle varies.”

### 4.3 Geometry

Part 3D solid geometry is provided in two file types in the “Part Geometry.zip” folder:

• Solidworks 2019 Part file (*.sldprt), and
• Stereolithography file (*.stl).

The Solidworks part file and STL file are oriented correctly with the machine coordinate system {A}. However, the *position* of each part in {A} (its location on the build substrate) is not incorporated in the part geometry files. The lower left corner, or -X and -Y corner of each part, is shown in Fig. 1 and defined by the commanded laser position (not the actual position, nor the edge of the part). These corner points for each part are the following: Part 1: (-6,11) mm, Part 2: (-2,2) mm, Part 3: (2, -7) mm, Part 4: (6, -16) mm.

### 4.4 Scan Parameters and XYPT Command Files

Note that G-code files, which were provided in [2] and used within the SimpleAM (SAM) build command generation software [3], are not provided in this dataset as they are not yet readily transferrable or interpretable by other AM machines. However, these files can be made available upon request to the authors.

**Fig. 7:**
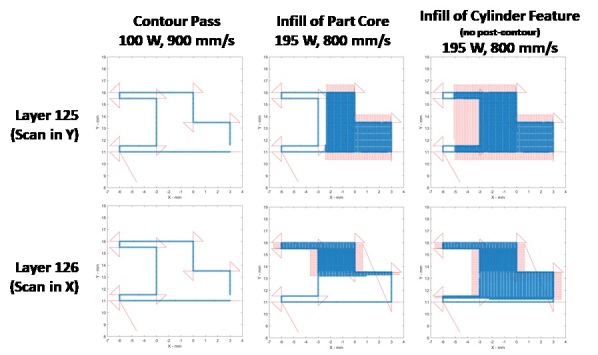
Example of scan strategy and *commanded* laser parameters for Part 1, layer 125 and layer 126. Red lines indicate laser is off, blue dots indicate laser is on. Note that *actual* position and laser power values are different and detailed in the Data Acquisition files (see Sec. 5.3).

Nominal scan conditions utilized primarily Y-direction scans for odd-numbered layers, and X-direction scans for even-numbered layers, as shown in Fig. 7. Commanded laser power was 100 W and scan speed 900 mm/s for a pre-contour pass, then infill with back-and-forth hatching occurred at commanded 195 W and 800 mm/s. The pre-contour pass is commanded at a position exactly on the part geometry edge, so it can be expected that the as-fabricated part would have dilated outer geometry that is (at least) ½ the melt pool width. The infill pattern covered the entire area of each part without being separated into sectors or stripes, and the hatch direction repeated for each part area within each layer. While scanning layers with the cylinder feature, the laser traversed across the cylinder cavity to cover each side, as shown in Fig. 7 - right. Traversal of the laser spot when the laser is off occurs at 800 mm/s. Turnaround time, or skywriting time, was 3 ms.

The *commanded* power, speed, or position is not the same as the *actual* power, speed, or position. Refer to Sec. 5.3 on the Data Acquisition files (DAQ), where actual feedback signals from the galvo scanner and laser power are described. True laser power is slightly less than commanded, and the positioning error of the galvo caused the laser spot to build parts slightly contracted in both the X and Y directions.

The full, unambiguous description of the laser parameters and scan strategy cannot be fully defined through a brief set of parameter and text description. The exact commanded laser power, speed, and position is encoded in the XYPT command files (which stands for X position, Y position, Power, and Trigger). The XYPT command files are stored in the “XYPT Commands.zip” folder. The following data description excerpt is taken from [2] which provided similar file structure, with filenames and figure references changed for this document:

“The files are formatted as comma-separated value (.CSV) American Standard Code for Information Interchange (ASCII) text in four columns, where each file named “XYPT_L0XXX.csv” provides the commands for layer number XXX.

**Fig. 8:**
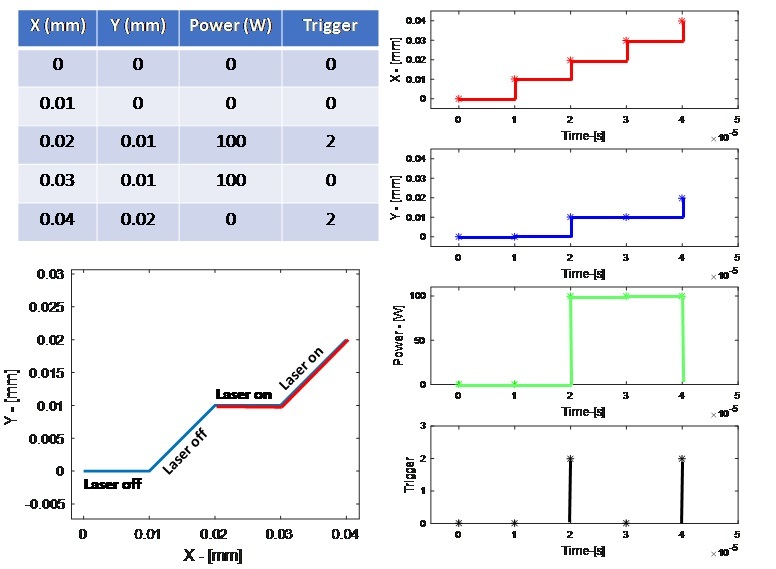
Demonstration of the XYPT files. Upper left: Four-column data and units in the XYPT files. Lower left: Schematic showing the resulting commanded XY position of the laser and when the laser turns on. Right: Plots of each individual column’s resulting effect on the AMMT. Note that the ‘trigger’ occurs for 20 μs, which is indicative of the camera integration time discussed in Sec. 5.1. Figure reproduced from [2].

*The XYPT files provide the basic laser positioning and control commands for the AMMT. They are based on the XY2-100 command protocol for laser/galvo systems* [3]. *Figure 8** gives an example of the XYPT structure, and the resulting laser/galvo command. Each row represents a 10 μs timestep, resulting in a 100 kHz digital command. The X (mm) and Y (mm) columns provide a position command for the laser/galvo. The power (W) column provides the laser power command.*

The trigger column (unitless) is a decimal representation of the binary number which is used to fire the output trigger channels (8 channels labeled channels 0 through 7). For example, to trigger channel 1 (the second channel), binary representation is 0010, decimal representation is ‘2’, and so a ‘2’ is in the ‘Trigger’ column in the XYPT file. To trigger both channel 0 and 2 (the first and third channels), binary representation is 0101, decimal representation is 5, and there would be a ‘5’ in the trigger column. For this dataset, only channel 1 is connected to the melt pool monitoring camera; therefore a ‘2’ in the T column indicates when a frame is captured.”

## 5 In-situ Process Monitoring Data

### 5.1 Co-axial Melt Pool Monitoring (MPM) Camera

The co-axial melt pool monitoring (MPM) camera is the same used in [2]. However, the frame rate was increased to a maximum of 10 000 Hz using a field-programmable gate array (FPGA) based acquisition. The camera is optically aligned with the laser, such that the field of view of the camera is fixed to the laser spot coordinate system {L} shown in Fig. 4, and the melt pool remains nominally stationary in the image. Table 1 provides operating characteristics of the MPM camera. The timing for each MPM image frame corresponds to a trigger point in the XYPT command files discussed in the previous section.

**Table 1:** Co-axial melt pool monitoring (MPM) camera parameters.

Camera Model	Mikrotron EOSens 3CL^1^
Pixel Pitch of Detector	8 μm
Window Size (H × V)	120 pixels × 120 pixels
Field of View (FoV)	0.96 mm × 0.96 mm
Instantaneous Field of View (iFoV)	8 μm/pixel
Viewing Angle	Same as laser position vector See Sec. 4.2.
Frame Rate	Up to 10 000 Hz
Integration Time / Shutter Speed	20 μs
Bit depth	8-bit (256 digital levels)
Optical Filter Bandwidth (Center ± FWHM bandpass)	850 nm ± 20 nm

Melt pool monitoring image files are stored in two file types. First, in the “Melt Pool Camera AVIs Lagarith.zip” file, 8-bit audio-visual interleave (AVI) video files are stored with the naming convention “MPMcamera_L0xxx.AVI”, where xxx is the layer number. Note that these video files are compressed using the open-source Lagarith lossless compression codec.

The other MPM camera file format is stored in the “Melt Pool Camera TIF Stacks.zip” file. This contains TIF image stack files with the naming convention “MPMcamera_L0xxx.TIF”, where xxx is the layer number. These 8-bit uncompressed TIF image stacks are singular files, where each image within the stack is a video frame. These were created and can be viewed using the open source image processing software ImageJ. Refer to the ImageJ “Stacks, Virtual Stacks, and Hyperstacks” online documentation.

**Fig. 9:**
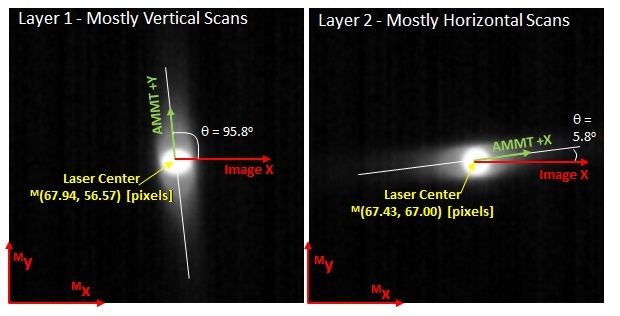
Orientation of machine coordinates {A} (AMMT +X and AMMT +Y in the image) within MPM camera image coordinates {M}, as well as laser center position, based on composite image of primarily vertical (left image) and horizontal (right image) scans.

Several important similarities and changes are made for the MPM images in this dataset compared to [2], and the authors suggest readers review that document for further details. First, MPM images, with coordinate system indicated by superscript {M}, are nominally oriented and aligned with the machine coordinate system {A} mentioned in Sec. 3.1. Therefore, the images do not need preprocessing transformations described in [2]. A new method was used to determine the relative orientation between MPM image coordinates in {M}, located at the lower-left of each image, and laser spot center, which defines the origin of {L} and can be located within in {M}, as shown in Fig. 9. All image frames for Layer 1 (left) and Layer 2 (right) were summed, respectively, to form a composite image. Laser spot center position was assumed as the center of mass of the composite image. Orientation was assumed to be along the calculated shape major axis defined by the best-fit ellipse, and shown as a white line in Fig. 9. Both center of mass and major axis were calculated using ImageJ’s “Analyze>Measure…” tools. This resulted in an orientation {M}→{A} of 5.8° CW, and laser spot center position in {M} of ^M^(67.94,56.57) pixels.

The calibration file “2019_MikrotronCal.csv” provides data that enables thermal calibration of the co-axial MPM images. This calibration enables mapping from camera signal in digital levels [DL] to apparent or radiance temperature in [°C], and enables 1) a verification of camera repeatability, 2) relation to other thermal imaging systems or camera operating parameters, and 3) potential computation of true surface temperature given a known or assumed surface emittance. Refer to [4, 5] for details on these calculations.

**Fig. 10:**
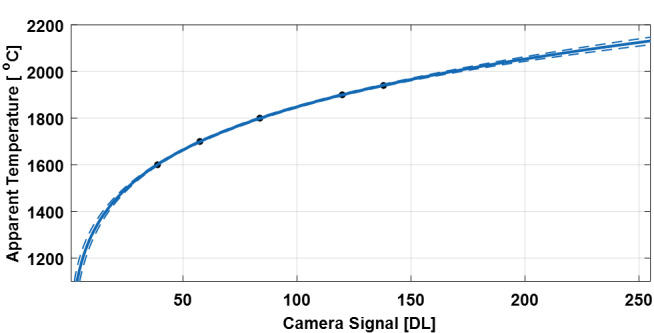
Thermal calibration of the co-axial MPM camera. Black dots are measurement points, and blue lines are the Sakuma-Hattori curve fit and 90% prediction intervals

Thermal calibration used a custom light emitting diode (LED)-driven integrating sphere source called the 850 nm thermal integrating sphere source (TISS850), which is calibrated against NIST primary standards. Five measurements were made while observing the TISS850 at different setpoint radiance temperatures, in which the signal over the 120 pixel by 120 pixel array was averaged. Figure 10 shows these points as black dots. To extrapolate the range of the calibration to temperatures above or below that obtainable with the TISS850, a calibration function called the Sakuma-Hattori equation shown in (1) was fit to these points, where *S* is camera signal in [DL], T_app_ is apparent or radiance temperature in [°C], c_2_ is the second radiation constant (14 338 µm/K), and A, B, and C are fit coefficients. Note that while T_app_ is given in [°C], the Sakuma Hattori equation is applied to temperature units of Kelvin.

$T_{app}\left(S\right)=\frac{c_{2}}{A\ln\left(\frac{C}{S}+1\right)}-\frac{B}{A}-273.15\;(1)$

Sakuma-Hattori curve fitting resulted in coefficients A = 0.2970599, B = 464.23278, C = 5.120179e+07. The calibration file “2019_MikrotronCal.csv” is provided which gives both the measured calibration values, and extrapolated values using Eq. (1).

### 5.2 Layer-wise Imaging Camera

**Fig. 11:**
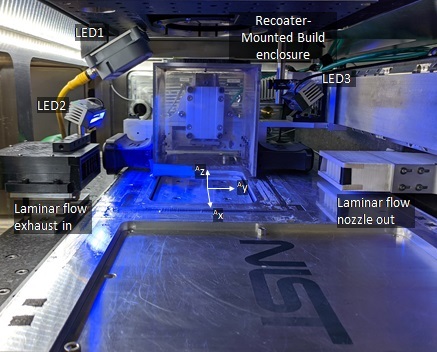
View inside AMMT chamber showing three illumination LED locations. Layer-wise imaging camera views the build plane from the window in the ceiling of the chamber.

The layer-wise imaging camera captured images of the build surface before and after recoating powder for each layer, as depicted in the experiment setup in Fig. 3. Multiple advancements were made since the publication of [2], including 1) change in illumination wavelength to blue 470 nm, 2) addition of three directional illumination sources, and 3) acquisition of images when the acrylic build enclosure is not above the build plate, removing the need to view through acrylic. Illumination consisted of one diffusely emitting brick LED array (LED1), and two linear LED arrays (LED2 and LED3). Figure 11 depicts the locations of the LED sources within the process chamber. Table 2 provides the camera parameters for the layer-wise imaging camera, as well as some information on the illumination sources.

**Table 2:** Layer-wise imaging camera parameters.

Camera Model	Basler acA3800-10gm^1^
Lens Model	Edmund Optics #58-000
Lens Focal Distance	8.5 mm
Pixel Pitch of Detector	1.67 μm
Window Size (H x V)	2000 pixels × 2000 pixels
Instantaneous Field of View (iFOV) (Approximate)	X: (63 to 71) μm/pixel Y: (58 to 63) μm/pixel See [2]
Viewing Angle (defined in {A})	^A^<0.45, 0, 0.89> 63.2° from surface normal
Frame Rate	6 per layer (before powder spreading and after)
Bit depth	12-bit
Optical filter on camera	Edmund Optics #89-764 470 nm central wavelength 85 nm FWHM
Illumination: LED 1	IVS Imaging SC75-470 470 nm central wavelength
LED2 and LED3	Prophotonix LN1-470-IXC100

**Fig. 12:**
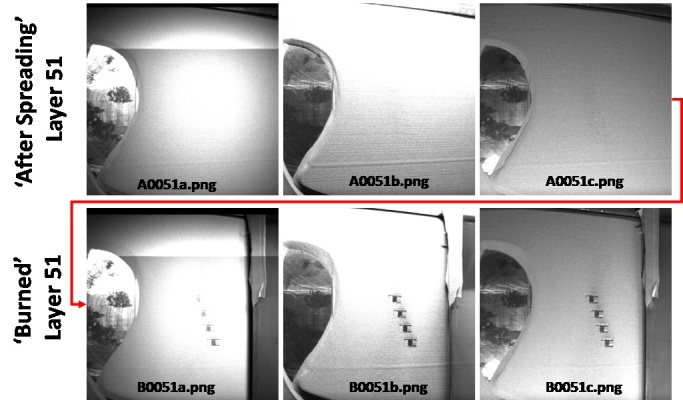
Example layer images and file naming convention. Six total pictures are taken per layer: Three illumination conditions after spreading, and three illumination conditions after laser exposure.

The layer-wise image file naming convention is similar to [2], and is based on whether images are captured after a layer of powder is spread (A for ‘after’, AxxxxY), or after the laser has melted that layer (B for ‘burned’, BxxxxY), where xxxx is the layer number, and Y indicates which LED source was illuminated when the image was taken (a, b, or c representing LED1, LED2, or LED3). This results in six images per layer. A simple way to remember: B0001a = ‘Burned layer 1, LED 1’ or A0002b = ‘After spreading layer 2, LED2’. Figure 12 gives an example sequence of images as they are captured in time, and their respective filenames. These image files are stored as portable network graphics (PNG) files in the folder “Layer Camera PNGs.zip”.

**Fig. 13:**
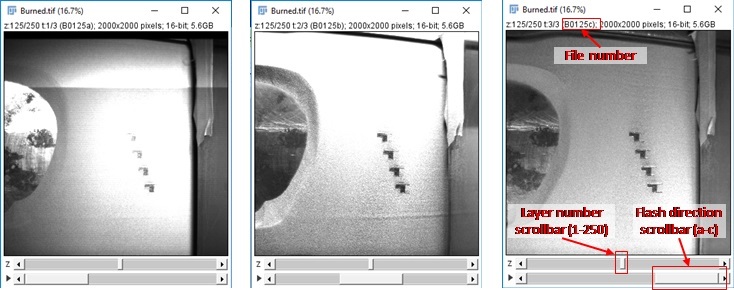
Example view of “Burned.tif” image stack in ImageJ showing how layer number and flash direction is set.

In addition to individual portable network graphics (PNG) images, layer images were compiled into TIF hyperstacks using the open source image processing software ImageJ. Refer to the ImageJ “Stacks, Virtual Stacks, and Hyperstacks” online documentation. The TIF hyperstacks are ordered “xyczt”, meaning that data is organized by ‘xy’ pixels, ‘c’ channels, ‘z’ slices (layer number) and 't' frames (LED number). The ‘c’ channel has no data and is therefore 1. Figure 13 shows an example of opening the “LayerCameraBurned.tif” hyperstack in ImageJ. Powder layer images are stored in “LayerCameraAfterSpreading.tif”.

In order to spatially calibrate, correct for perspective or other distortion, and ultimately register the layer camera images coordinates {C} with the machine coordinate system {A}, a series of additional measurements were made. The following calibration and registration metadata are provided in the “Layer Camera Metadata.zip” folder:

• DotGrid_2000x2000.tif:
  o Dot grid calibration target imaged with the layer camera.
• Checkerboard_2000x2000.tif:
  o Checkerboard calibration target imaged with the layer camera. This target is nominally positioned with the machine ^A^(0,0) origin, and oriented with ^A^(x,y) axes, but not precisely.
• SecondaryCamera_Laser00.tif:
  o Image of dot grid target taken with a second camera (not the layer camera), while a red laser indicator is positioned at ^A^(0,0).

Both dot grid and checkerboard patterns are provided since various image processing software applications have utilities that recognize either. For example, LabVIEW VisionAssistant calibrates using dot grids for automated image spatial calibration and distortion correction, and the Matlab Computer Vision Toolbox utilizes checkerboard targets for the same. The dot grid is an Edmund Optics #59-211, with dots of 0.5 mm diameter spaced 1.00 mm apart on a 50×50 grid. The checkerboard target is an Edmund Optics #12-202, 1.4 mm × 1.4 mm squares.

Registration of layer camera image coordinates {C} with machine coordinates {A} can be accomplished two ways: 1) Use the ‘Checkerboard_2000x2000.tif’ image as a template to remove distortion in the layer images, and set the machine coordinates based on the central feature, which is nominally aligned and centered with the machine coordinates ^A^(x,y); 2) Use the ‘SecondaryCamera_Laser00.tif’ image, and locate the machine coordinate center ^A^(0,0) with respect the dot grid based on the location of the red dot, then, use ‘DotGrid-2000x2000.tif’ image for distortion correction, and assign the machine coordinates based on the known location of the laser center with respect to the laser dot.

**Fig. 14:**
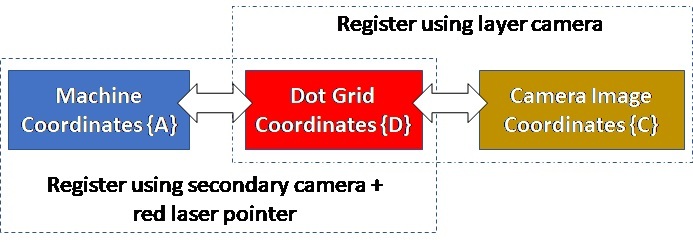
Schematic describing use of secondary camera and dot grid to location machine coordinates within the layer camera images.

**Fig. 15:**
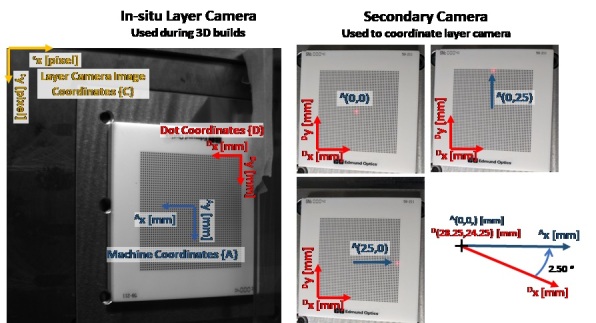
Demonstration of method used for determining orientation of the dot grid, indicated by coordinate system {D}, with respect to the AMMT machine or base coordinate system {A} shown in Fig. 4.

A schematic of method #2 is shown in Fig. 14, which shows how machine coordinates can be mapped to the layer camera image, or vice versa, using the secondary camera image. While this provides the machine coordinate origin ^A^(0,0) in the layer camera images, it does not give the orientation. Furthermore, the dot grid target was not physically aligned with the machine coordinates. Additional measurements were taken with the secondary camera and red laser dot positioned at various points on the dot grid. These points were extracted by color thresholding the images, then mapped to a coordinate system assigned to the dot grid, {D}. The dot grid coordinate origin ^D^(0,0) aligns with the center of the lower-left dot. The orientation of the dot grid {D} with respect to the machine coordinates {A}, was then determined to be 2.5° as shown in Fig. 15. The machine coordinate origin in dot grid coordinates is ^A^(0,0) = ^D^(28.25, 24.25) mm.

### 5.3 Data Acquisition (DAQ) data

In addition to the MPM camera and the layer-imaging camera, the AMMT incorporates an analog signal data acquisition system. The analog to digital conversion sampling rate is 100 kHz. This was used to monitor four channels: 1) Galvo X-mirror encoder, 2) Galvo Y-mirror encoder, 3) the LTZ lens encoder (see Fig. 3), and a laser power reference signal from the laser console. While XYPT files mentioned in Sec. 4.4 provide the commanded laser power and scan path, the DAQ data provides the *actual* laser power and scan path, provided there will be uncertainties stemming from the DAQ calibration. Refer to [6] for a detailed description of the galvo scanner and its encoder calibration, and [7] for a detailed description of the laser power and power reference signal calibration.

DAQ files are provided in “DAQ Data.zip”. The files have the naming convention “DAQ_L0xxx.txt”, where xxx indicates the layer number. The files are tab-delimited text files with 29 lines of header information, as shown in Fig. 16. The ‘Calibration Polynomials’ header section stores the calibration for each channel, which converts from the measured analog signal [V], to the unit of interest (mm), W,, using a polynomial of the form y = a_0_+a_1_x+a_2_x^2^+…+ a_7_x^7^,… where x is measured analog voltage [V], and y is the unit of interest. The data for each channel, collected at 100 kHz such that each row occurs in 10 μs intervals, is provided in tab-delimited columns starting on line 30.

**Fig 16:** Example first header and data lines of a tab-delimited DAQ data file. Channel data starts at line 30. **Table:** 

1 2 3 4 5 6 7 8 9 10 1 12 13 14 15 16 17 18 19 20 21 22 23 24 25 26 27 28 29 30 31 32 33	Additive Manufacturing Metrology Testbed DAQ Data Export File --- This file is TAB-DELIMITED. Be careful if editing this header! Some strings here may be used for automated parsing. Source Filename: Full Source File Path: File Export Timestamp (NOT experiment timestamp): 3/31/2020 12:01:53 PM Number of Channels: 4 Number of Rows: 409600 --- Calibration Polynomials Coeff# CH0 CH1 CH2 CH3 CH4 CH5 CH6 CH7 CH8 Coeff0 0.801600 6.318600 2.717000 0.000000 -6.55560 0.000000 0.000000 0.000000 0.000000 Coeff1 -30.9037 36.84810 1.396000 1.000000 96.42240 1.000000 1.000000 1.000000 1.000000 Coeff2 0.000000 0.000000 0.000000 0.000000 0.000000 0.000000 0.000000 0.000000 0.000000 Coeff3 0.000000 0.000000 0.000000 0.000000 0.000000 0.000000 0.000000 0.000000 0.000000 Coeff4 0.000000 0.000000 0.000000 0.000000 0.000000 0.000000 0.000000 0.000000 0.000000 Coeff5 0.000000 0.000000 0.000000 0.000000 0.000000 0.000000 0.000000 0.000000 0.000000 Coeff6 0.000000 0.000000 0.000000 0.000000 0.000000 0.000000 0.000000 0.000000 0.000000 Coeff7 0.000000 0.000000 0.000000 0.000000 0.000000 0.000000 0.000000 0.000000 0.000000 --- Data begins below. First header line is the channel index. Second header line is the channel's assigned label. Third header line is the channel's assigned units. --- CH0 CH1 CH2 CH4 X Y LTZ P mm mm mm W -0.02915 -0.02071 0.166471 -0.527361 -0.01854 -0.01312 0.166375 -0.825297 -0.02809 -0.00679 0.166040 -0.106940 -0.03976 0.008385 0.166759 0.091684

**Fig. 17:**
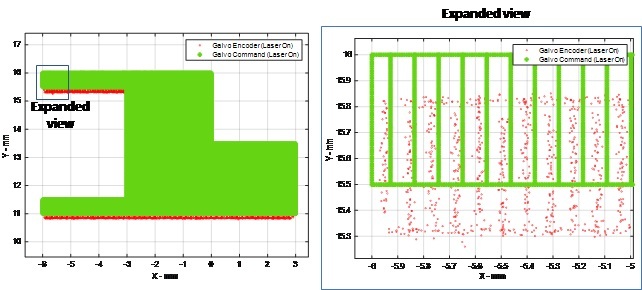
Example plot of Part 1, Layer 125, comparing commanded galvo position from XYPT files vs. galvo encoder readout from DAQ data.

**Fig. 18:**
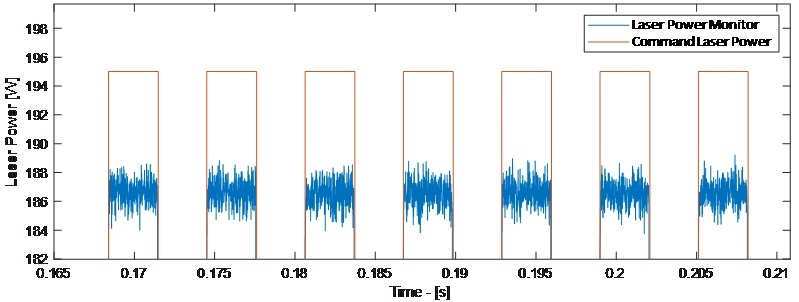
Example plot of Part 1, Layer 125 comparing commanded laser power from XYPT files vs. power monitoring signal from DAQ data.

An example is given in Fig. 17 that compares the commanded galvo position, based on values from the XYPT command data, versus the galvo encoder data, provided in the DAQ data. This data is plotted for only those points when the laser is either commanded on (in XYPT data), or measured on (in DAQ data). Two things can be observed: the galvo encoder data exhibits some inherent noise, and encoder-measured galvo positions show error compared to commanded positions. This positioning error demonstrates the ‘pre-compensation’ galvo calibration described in [6], which was corrected and improved after this 3D build. Similarly, Fig. 18 demonstrates a comparison between commanded laser power and monitored laser power. The monitored laser power, provided in the DAQ data, exhibits some noise as well as indicating an erroneously low applied power. This laser power calibration is exemplified by the ‘pre-compensation’ measurements described in [7].

## 6 Data Files

The dataset contains the following files and file structure, where sub-folders are indicated by a ‘>’ symbol:

• Lane-JResData-OverhangPartX4.pdf
  o This dataset overview document.
• Build Metadata.zip >
  o Powder>
    ▪ Powder_CharX.txt: Text file with powder size distribution measurements taken from three samples (X = 1,2,3).
    ▪ PowderParametersInfo.docx: MS Word document with description and mathematical definition of various powder particle size metrics given in “Powder_CharX.txt”
  o 2018_AMMTLaserScanAngles.txt: A tab-delimited text file that provides the laser incident angle unit vectors with respect to the build plane as shown in Fig. 4.
  o LaserSpotMeasure70x70.tif: Tagged image format image file representing the measured laser spot energy distribution in Fig. 5. Pixel values are relative and unitless.
• Part Geometry.zip >
  o OverhangPart_9x5x5mm.SLDPRT: Solidworks 2019 part file
  o OverhangPart_9x5x5mm.STL: Stereolithograpy file of the individual overhang part.
• XYPT Commands.zip >
  o XYPT_L0xxx.csv: Comma-separated text files containing build command data. L0xxx represents 3D build layer number xxx. Each row represents a 10 μs interval.
• Layer Camera Metadata.zip >
  o DotGrid_2000x2000.tif: 8-bit grayscale tagged image format file of the dot grid calibration target taken with the layer camera.
  o SecondaryCamera_Laser00.tif: 24-bit color (RGB) tagged image format file of the dot grid calibration target taken with the secondary camera, including a red dot indicating the machine coordinate center ^A^(0,0).
  o Checkerboard_2000x2000.tif: 8-bit grayscale tagged image format file of the checkerboard calibration target taken with the layer camera.
• Layer Camera_PNGs.zip >
  o AxxxxY.png: 16-bit grayscale portable network graphics image file taken with the layer camera during the 3D build after a new layer of powder is spread, where xxxx indicates the layer number, and Y = a,b, or c is the LED illumination number for that image.
  o BxxxxY.png: 16-bit grayscale portable network graphics image file taken with the layer camera during the 3D build after the laser has melted the powder layer, where xxxx indicates the layer number, and Y = a,b, or c is the LED illumination number for that image.
• LayerCameraAfterSpreading.tif: Tagged image format hyperstack. Contains the same image data as AxxxxY. Hyperstack is ordered “xyczt”, meaning data is organized by ‘xy’ pixels, ‘c’ channels, ‘z’ slices (layer number) and 't' frames (LED number).
• LayerCameraBurned.tif: Tagged iamge format hyperstack. Contains the same image data as BxxxxY. Hyperstack is ordered “xyczt”, meaning data is organized by ‘xy’ pixels, ‘c’ channels, ‘z’ slices (layer number) and 't' frames (LED number).
• Melt Pool Camera Metadata.zip >
  o 2019_MiktrotronCal.csv: Comma separated text file with measured radiance temperature vs. camera signal values, and extrapolated radiance temperature vs. camera signal values.
• Melt Pool Camera AVIs Lagarith.zip >
  o MPMcamera_L0xxx.AVI: 8-bit audio-visual interleave video files, where ‘xxx’ indicates the layer number. Files are encoded using the Lagarith lossless codec. Each frame corresponds to a nonzero value in the ‘T’ column of the XYPT command files.
• Melt Pool Camera TIFF Stacks.zip >
  o MPMcamera_L0xxx.tif: 8-bit tagged image file stack, where xxx indicates the layer number. Each image in the stack represents one video frame which corresponds to a nonzero value in the ‘T’ column of the XYPT command files.
• DAQ Data.zip >
  o DAQ_L0xxx.txt: Tab-delimited text file representing four channels measured from the data acquisition system. Data starts on line 30, and each row indicates a time step of 10 μs.

## 7 Impact

This dataset provides open access, industrially-relevant AM in-situ process monitoring data for use with various image or data processing or machine learning algorithms. This process monitoring data is otherwise inaccessible on commercial AM machines, or lacks key calibration or characterization metadata as provided here. This dataset is part of the NIST Metrology for Real-Time Monitoring of Additive Manufacturing project.
